# Utilizing Liposomal Quercetin and Gallic Acid in Localized Treatment of Vaginal *Candida* Infections

**DOI:** 10.3390/pharmaceutics12010009

**Published:** 2019-12-20

**Authors:** Barbara Giordani, Purusotam Basnet, Ekaterina Mishchenko, Barbara Luppi, Nataša Škalko-Basnet

**Affiliations:** 1 Department of Pharmacy and Biotechnology, University of Bologna, Via San Donato 19/2, 40127 Bologna, Italy; barbara.giordani4@unibo.it (B.G.); barbara.luppi@unibo.it (B.L.); 2 Drug Transport and Delivery Research Group, Department of Pharmacy, Faculty of Health Sciences, University of Tromsø The Arctic University of Norway, Universitetsveien 57, 9037 Tromsø, Norway; 3 IVF Clinic, Department of Obstetrics and Gynecology, University Hospital of North Norway, Sykehusvegen 38, 9019 Tromsø, Norway; purusotam.basnet@uit.no; 4 Women’s Health and Perinatology Research Group, Department of Clinical Medicine, University of Tromsø The Arctic University of Norway, Universitetsveien 57, 9037 Tromsø, Norway; 5 Department of Medical Biology, Faculty of Health Sciences, University of Tromsø The Arctic University of Norway, Sykehusveien 44, 9037 Tromsø, Norway; ekaterina.mishchenko@uit.no

**Keywords:** vaginal infection, liposomes, *Candida*, polyphenols, quercetin, gallic acid

## Abstract

Vulvovaginal candidiasis (VVC) is a widely spread fungal infection that causes itching, pain and inflammation at the vaginal site. Although common, currently available treatment suffers from limited efficacy and high recurrence. In addition, the growing problem of resistance to azole drugs used in current treatments emphasizes the need for superior treatment options. Antimicrobial polyphenols are an attractive approach offering multitargeting therapy. We aimed to develop novel liposomes for simultaneous delivery of two polyphenols (quercetin, Q, and gallic acid, GA) that, when released within the vaginal cavity, act in synergy to eradicate infection while alleviating the symptoms of VVC. Q was selected for its anti-itching and anti-inflammatory properties, while GA for its reported activity against *Candida*. Novel liposomes containing only Q (LP-Q), only GA (LP-GA) or both polyphenols (LP-Q+GA) were in the size range around 200 nm. Q was efficiently entrapped in both LP-Q and in LP-Q+GA (85%) while the entrapment of GA was higher in LP-Q+GA (30%) than in LP-GA (25%). Liposomes, especially LP-Q+GA, promoted sustained release of both polyphenols. Q and GA acted in synergy, increasing the antioxidant activities of a single polyphenol. Polyphenol-liposomes were not cytotoxic and displayed stronger anti-inflammatory effects than free polyphenols. Finally, LP-GA and LP-Q+GA considerably reduced *C. albicans* growth.

## 1. Introduction

Vulvovaginal candidiasis (VVC) is an ever-living problem affecting 70–75% of women of reproductive age at least once during their life. Around 40–50% of them will experience a recurrence, and especially serious is VVC among pregnant women. *Candida albicans*, and other non-*albicans* related species, are the major causative agents of VVC [1,2]. Although *C. albicans* is a commensal microorganism, the perturbation of vaginal homeostasis could facilitate the overgrowth of this opportunistic fungus and the onset of symptomatic candidiasis. Even if VVC is not a life-threatening problem, it can impair the quality of life of patients leading to several physical and sexual impediments, with economic costs estimated at one billion dollars per year [1,2,3]. Candidiasis clinically manifests mostly through itching, pruritus, irritation, burning, pain, white cheesy discharges, vulvar and vaginal erythema, and edema [4].

Fidel et al. [5] proved that the pathogenesis of VVC has a prominent immunological component, involving the recruitment of neutrophils to the vaginal mucosa and activation of related proinflammatory cytokine and chemokines. This strong inflammatory response limits possibilities to control the *Candida* burden, exacerbates the tissue damage and discomfort, and can trigger chronic infections [4].

Therapeutic approaches for VVC comprise both local and oral administration of different azole drugs, such as fluconazole, ketoconazole and clotrimazole [6], that are able to reduce symptoms of an initial infection in a large number of patients [3]. Unfortunately, since all azoles have a similar fungistatic effect on *Candida* spp., the cells exposed repetitively to these antifungals may adapt to drug pressure and became resistant [7]. Taking into consideration the emerging problem of drug resistance, as well as the high incidence of VVC, it is clear that new therapeutic strategies based on both administration of alternative antimicrobials and development of advanced delivery systems, are extremely desirable.

Among natural molecules, polyphenols have gained increasing interest in the last few years as potential candidates for *Candida* treatment [8,9]. Polyphenols are phytochemicals that can be principally found in cereals, grains, legumes, fruits, vegetables and in beverages, e.g., tea, coffee, fruit juice and cocoa [10]. Based on their chemical structure, polyphenols can be categorized into four main groups: flavonoids, stilbenes, lignans and phenolic acids. In humans, besides the well-established antioxidant activity, polyphenols display many other biological effects, being able to act as anti-inflammatory, antidiabetic, cardioprotective, antiaging [11] and antimicrobial agents [12,13].

In the present work, we have, for the first time, utilized quercetin (Q) and gallic acid (GA) as polyphenols of interest in topical therapy of vaginal *Candida*. Quercetin (3,3′,4′,5,6-pentahydroxyflavone) is the most potent antioxidant among polyphenols that has been proposed for the treatment of a wide spectrum of pathologies, including diabetes, circulatory dysfunctions and cancers [14]. The therapeutic effects of quercetin mainly depend on its abilities to scavenge reactive oxygen radicals such as O_2_^−^ and ONOO^−^, protect from lipid peroxidation and chelate metal ions. In addition, its inhibitory effects on cytokine production (i.e., TNF-α and IL-8) and histamine release [15] lead to a reduction of the inflammation state. Moreover, several groups [16,17] have also suggested that quercetin may be an antinociceptive in animal models, thus relieving the pain associated with inflammation. Interestingly, Maramaldi et al. [18] demonstrated that phytosomes containing quercetin were able to exert a lenitive and anti-itch effect in a group of volunteers with skin damage. Gallic acid (3,4,5-trihydroxybenzoic acid) shares with quercetin some important biological features, mostly imparted by the hydroxyl group. It is largely studied for its strong antioxidant and anti-inflammatory properties, as well as anti-carcinogenesis and anti-arteriosclerosis effects. Furthermore, gallic acid is reported to inhibit microbial biofilm formation, possess bactericidal effects towards both Gram-positive and Gram-negative bacteria and exhibit antiviral activities against a human immunodeficiency virus [19]. Several authors proposed that gallic acid is also able to exert an antifungal activity against planktonic cultures and biofilm of *C. albicans* [12,13,20]. Moreover, Li et al. [21] demonstrated that gallic acid inhibited the growth of different clinical isolates of *Candida* spp. and proposed that the fungicidal outcome was due to the impairment of biosynthesis of ergosterol, a fundamental component of the fungal membrane.

Despite the favorable pharmacological properties of polyphenols, their applicability remains limited by their low solubility and bioavailability as well as high susceptibility to environmental conditions, including biological environment. In this regard, the incorporation of polyphenols inside a nanocarrier can be an appropriate approach to avoid the degradation of active molecules and promote their deposition at the site of administration assuring enhanced biological effect [22,23,24].

In this work, a novel liposomal system for the simultaneous delivery of quercetin and gallic acid to the vaginal cavity was developed. The combination of quercetin and gallic acid was chosen with the aim of achieving a double effect, namely the immediate and long-lasting alleviation of VVC symptomatology and the eradication of the fungus infection. The purpose was to obtain a formulation able to guarantee a modified release of both active compounds, thus assuring adequate concentration of polyphenols in the vaginal mucosa. Liposomes are vesicular nanostructures composed of one or more lipid bilayers, and are particularly suitable for the combined delivery of two molecules with different physicochemical properties since they are able to accommodate both the lipophilic compounds, such as quercetin, and more hydrophilic substances, such as gallic acid, inside the lipid and aqueous compartments, respectively [24,25].

Liposomal formulations were characterized for their technological (size, zeta potential, entrapment efficiency, release behavior and stability over time) and biological properties (antioxidant and anti-inflammatory activities and cytotoxicity). Finally, the capability of proposed formulations to exert an antifungal effect against *Candida* was also investigated.

## 2. Materials and Methods

### 2.1. Materials

Lipoid S 100 from fat-free soybean lecithin, containing not less than 94% phosphatidylcholine (SPC), was a kind gift from Lipoid GmbH (Ludwigshafen, Germany). Quercetin (Q, Mw: 302.2, logP: 2.16, purity ≥95%), gallic acid (GA, Mw: 188.1, logP: 0.7 purity ≥99%), potassium phosphate monobasic, propylene glycol (PG), 2,20-azino bis(3-ethylbenzothiazoline)-6-sulfonic acid diammonium salt (ABTS), 1,1-diphenyl-2-picrylhydrazyl (DPPH), vitamin C (ascorbic acid), vitamin E (α-tocopherol), RPMI 1640 medium, bovine serum albumin (BSA), glutamine and Cell Counting Kit-8 (CCK-8) were purchased from Sigma-Aldrich, Chemie GmbH (Steinheim, Germany). Lipopolysaccharide (LPS; *Escherichia coli*, 055:B5), sulfanilamide, naphthylethylenediamine dihydrochloride, phosphoric acid and sodium nitrite were acquired from Sigma Life Science (Sigma-Aldrich Norway AS, Oslo, Norway). Murine macrophage RAW 264.7 cell line and *Candida albicans* strain (ATCC 10231) were supplied by ATCC (Manassas, VA, USA).

All solvents were of analytical grade and were provided by VWR International bvba/sprl (Leuven, Belgium).

*Candida* was grown in potato dextrose broth (Sigma-Aldrich Co., St. Louis, MO, USA) with 2% of glucose (PDB_Glu_) or on agar plates of the same medium (PDA_Glu_).

Buffer solution at pH 4.5 simulating vaginal pH (3.5–5.5) was prepared as follows: KH_2_PO_4_ 0.1 M. The composition of Griess reagent was: sulphanilamide 1%, naphthylethylenediamine 0.1% dihydrochloride, phosphoric acid 2.5%.

### 2.2. Preparation of Liposomes

The Q and GA co-loaded liposomes (LP-Q+GA) were prepared by a film hydration method as previously described [25], with some modifications. Briefly, Q (10 mg) and SPC (200 mg) were dissolved in 10 mL of methanol in a round-bottom flask. The solvent was removed by evaporation (Büchi rotavapor R-124 with vacuum controller B-721, Büchi Vac^®^ V-500, Büchi Labortechnik, Flawil, Switzerland) under vacuum (45 mmHg) for 2 h at 50 °C. The resulting dry lipid film was rehydrated with a solution of GA in distilled water (1 or 2 mg/mL), obtaining multilamellar vesicles (MLV). Liposomes containing only Q (LP-Q) or GA (LP-GA) were prepared under the same conditions by adding only Q (10, 15 or 20 mg) in the methanol lipid solution or dissolving GA in the aqueous phase (1 or 2 mg/mL), respectively. Plain liposomes (plain-LP) were prepared using only SPC (200 mg). All liposomal dispersions were stored at 4–8 °C overnight.

### 2.3. Size Reduction of Liposomes

Sonication was used to obtain liposomes of the desired size [26]. The sonicator (Ultrasonic processor 500 W, Sigma-Aldrich, St. Louis, MO, USA) was set to 40% amplitude and ultrasonic irradiation for 4 cycles (30 s on/60 s off) was applied to liposomal dispersions. An ice bath was used to prevent heating of the dispersions. All sonicated liposomal formulations were stored at 4–8 °C.

### 2.4. Determination of Vesicle Size Distribution

The particle size and polydispersity index (PDI) of liposomal preparations were determined by photon correlation spectroscopy (submicron particle sizer model 370, Nicomp, Santa Barbara, CA, USA). To prevent interference from dust particles, the preparative procedure was carried out in a laminar airflow bench, as previously described [27]. Small aliquots of liposomal dispersions were diluted with filtered distilled water (0.22 μm syringe filter, VWR International, Leuven, Belgium) to obtain a particle count range of 250–350 kHz. All measurements were run (run time of 10 min) in the vesicle mode and intensity-weight distribution at room temperature (24–25 °C).

### 2.5. Zeta Potential

Zeta potential analysis was performed using a Malvern Zetasizer Nano-ZS (Malvern, Oxford, UK) [28]. Measurement cells (DTS1060) were rinsed with ethanol and filtered water (0.22 μm syringe filter) before loading the sample. Liposomal dispersions were diluted 1:20 (*v*/*v*) with filtered water to achieve an attenuation value of 6–7. Measurements were made at 25 °C with an equilibration time of 180 s and number of runs set to automatic.

### 2.6. Determination of Polyphenol Entrapment Efficiency

Since Q and GA display very different water solubility (the experimentally determined values were 4.1 ± 0.3 μg/mL and 13.7 ± 0.2 mg/mL, respectively), different protocols were applied for the determination of their entrapment efficiency.

Therefore, the entrapment efficiency of quercetin (Q EE %) in LP-Q and LP-Q+GA was determined after mild centrifugation (3000× *g*, 15 min, room temperature; Biofuge stratos centrifuge, Heraeus instruments GmbH, Hanau, Germany) to precipitate non-encapsulated Q. Then, Q was quantified by UV–visible spectroscopy (Spark Multimode Microplate Reader, Tecan^®^ Trading AG, Männedorf, Switzerland) at 372 nm both in the supernatants containing quercetin incorporated inside vesicles, and in not-centrifuged liposomes after dilution with methanol. The standard curve of Q in methanol was plotted at a concentration range of 2.5–37.5 μg/mL (R^2^ = 0.9997).

Considering the good solubility of GA in distilled water, to determine its entrapment efficiency (GA EE %) in LP-GA and LP-Q+GA, liposomes were separated from supernatant (containing freely unentrapped polyphenol) by ultracentrifugation (92,000× *g*, 1 h, 10 °C; Beckman model L8-70M with SW 60 Ti rotor, Beckman Instruments, Brea, CA, USA). Aliquots of pellet and supernatant, as well as not-centrifuged liposomes, were diluted with methanol and GA was quantified in each fraction by UV-visible spectroscopy (Spark Multimode Microplate Reader, Tecan, Männendorf, Switzerland) at 272 nm. The standard curve of GA in methanol was set up in the 2.5–37.5 μg/mL range (R^2^ = 0.9996).

### 2.7. Evaluation of Liposomes Storage Stability

Samples were stored at 4–8 °C and kept out of light by applying aluminum foil. The stability of prepared liposomes was evaluated by measuring their mean diameter, PDI, zeta potential and entrapment efficiency for Q and GA after 15, 30, 60 and 90 days. Four different batches of each formulation were analyzed in triplicate.

### 2.8. In Vitro Polyphenol Release Studies

The in vitro polyphenol release studies were performed by using Franz diffusion cells (1.77 cm^2^ diffusion area, 12 mL acceptor volume; PermeGear, Bethlehem, PA, USA) [29] equipped with a V6A Stirrer (PermeGear, Bethlehem, PA, USA). The heating circulator (Julabo Laboratechnik, F12-ED, Seelbach, Germany) was set to maintain a temperature of 37 °C. To mimic the vaginal pH [30], the acceptor chambers were filled with phosphate buffer pH 4.5 and ethanol was added at the final concentration of 20% (*v*/*v*) [31,32,33] to ensure sink conditions during the study (the solubility of Q and GA in the releasing conditions was found to be 0.226 ± 0.042 mg/mL and 78.7 ± 3.4 mg/mL, respectively).

Dialysis membranes (Mw cut-off: 12,000–14,000 Daltons; Medicell International Ltd., London, UK) pre-soaked in phosphate buffer pH 4.5 were fixed between donor and acceptor compartments [34]. LP-Q, LP-GA and LP-Q+GA were added to the donor chamber (500 μL). Solutions of Q (in PG solution 50%, *w*/*v*) and GA (in distilled water) at the same concentrations as in liposomes were used as controls.

Samples (500 μL) were withdrawn from the receptor chamber every hour for 8 h and immediately replaced by an equal amount of fresh medium. The amount of Q and GA was quantified spectrophotometrically at 372 nm and 260 nm, respectively. Calibration curves for Q (R^2^ = 0.9998) and GA (R^2^ = 0.9993) in releasing medium were obtained within the concentration range 2.5–25 μg/mL. Experiments were performed in triplicate and results are expressed as a cumulative percentage of polyphenols released over time.

### 2.9. Antioxidative Assays

The in vitro antioxidant activity of polyphenols formulated inside liposomes was assessed by employing two different methods, namely ABTS and DPPH assay [35].

LP-Q, LP-GA and LP-Q+GA and plain-LP were serially diluted in ethanol and tested at various SPC concentrations (10–600 μg/mL), corresponding to Q and GA concentrations of 0.5–30 μg/mL. The activity levels of vitamin E and C were also tested under the same experimental conditions and used as controls for comparison.

#### 2.9.1. ABTS**·**^+^ Radical Scavenging

ABTS**·**^+^ radicals were generated by mixing equal volumes (3 mL) of the stock solutions of ABTS (7.4 μM) and potassium peroxodisulphate (2.6 μM) in distilled water at room temperature [24]. The reaction mixture was allowed to stabilize for 18 h and then diluted with 100 mL of ethanol. A total of 300 μL of ABTS**·**^+^ radicals working solution was then added to equal volume of samples, shaken vigorously and kept in the dark for 30 min at room temperature. Reduction of blue-green colored radical solution by hydrogen-donating anti-oxidant was measured spectrophotometrically at 751 nm (Spark Multimode Microplate Reader, Tecan^®^ Trading AG, Männedorf, Switzerland).

#### 2.9.2. DPPH Radical Scavenging

DPPH radical scavenging activity was determined as reported by Jøraholmen et al. [24]. A total of 300 μL of ethanolic DPPH solution (60 μM) was thoroughly mixed with equal volume of each sample and kept in the dark for 30 min at room temperature. The disappearance of violet color after incubation time indicated a high free radical scavenging activity, spectrophotometrically quantifiable at 519 nm (Spark Multimode Microplate Reader, Tecan^®^ Trading AG, Männedorf, Switzerland).

Experiments were performed in triplicate and the radical scavenging activity (RSA) was calculated according to Equation (1):

RSA (%) = (1 − A_sample_/A_control_) × 100 (1)

where A_sample_ is the absorbance of the sample and A_control_ is the absorbance of the control samples prepared by mixing the same amount of ethanol and ABTS**·**^+^ or DPPH solution. Ethanol was used as a blank sample.

The combined effect of Q and GA in liposomes was investigated according to Loewe additivity model [36] and the interaction index (γ) was calculated (Equation (2)) as follows:

γ = d_1_/D_1_ + d_2_/D_2_ (2)

where D_1_ and D_2_ are the doses of Q (alone) and GA (alone) that have the RSA value of 50 (EC_50_) while d_1_ and d_2_ are the doses of Q and GA in the mixture that elicit the same effect.

γ > 1, =1 and <1 mean that the combination effect is antagonism, additive and synergy, respectively.

### 2.10. Cell Culture

The murine macrophage RAW 264.7 cell line was used to investigate a possible cytotoxic effect of free and formulated polyphenols and their ability to exert an anti-inflammatory activity. Cells were maintained in RPMI 1640 medium supplemented with BSA 10%, streptomycin 100 μg/L, and penicillin 100 IU/mL at 37 °C in a 5% CO_2_ atmosphere.

### 2.11. In Vitro Cell Viability Study

To assess the in vitro toxicity, RAW 264.7 cells, grown as described in Section 2.10, were seeded (90 μL) in 96-well flat bottom plates at the density of 5 × 10^4^ cells/well. The plates were pre-incubated for 24 h at 37 °C in 5% CO_2_ to allow the cells to stabilize. The adherent cells were then treated with 10 μL of media only (negative control) or liposomal formulations (LP-Q, LP-GA and LP-Q+GA) and subsequently incubated for 24 h. In particular, liposomes were diluted in the growth medium and tested at three different final SPC concentrations, namely 1, 10 and 50 μg/mL [28], corresponding to Q and GA concentrations of 0.05, 0.5 and 2.5 μg/mL, respectively. Solutions of Q and GA (alone or in combination) were prepared in PG, diluted in growth medium and tested at the same concentrations as in liposomal formulations. Plain-LP, at the same SPC concentrations as in loaded liposomes, was also evaluated. Distilled water and PG solution (added at the final concentration of 0.25% *w*/*v*, corresponding to the maximum volume of PG applied to cells) served as control.

After incubation, living cells were quantified using the Cell Counting Kit-8 (CCK-8), following the manufacturing instructions. Briefly, 10 μL of CCK-8 were added to the cells and the absorbance was measured at 450 nm (Spark Multimode Microplate Reader, Tecan^®^ Trading AG, Männedorf, Switzerland) after 4 h at 37 °C.

Since slight spontaneous absorbance may occur in a culture medium incubated with CCK-8, growth medium without cells was used as a blank. All experiments were performed in triplicate and results were expressed as percentage of living cells with respect to control (untreated cells).

### 2.12. Anti-Inflammatory Activity Determination

The capability of liposomes to inhibit nitric oxide (NO) production in LPS-induced macrophages was evaluated as previously described and expressed as anti-inflammatory activity [37].

Cells were cultured in 24-well plates (5 × 10^5^ cells/mL) for 24 h (Section 2.10). Old medium was then removed and replaced with fresh RPMI 1640 medium (1 mL) containing LPS (1 μg/mL) to induce NO production. Cells were treated with 10 μL of liposomal formulations or solutions at the same concentrations as applied in toxicity assay (Section 2.11), and incubated for 24 h. For a negative control, cells were treated with only LPS. The NO production by macrophages was correlated to nitrite formation in the media which was quantified by Griess methods. Equal volume of media and Griess reagent (300 μL) were mixed and incubated for 30 min. The absorbance was determined at 550 nm (Agilent Technologies, Santa Clara, CA, USA) and a calibration curve (0.5–20 μM) was constructed by using NaNO_2_ as standard with Griess reagent.

All experiments were performed in triplicate and the anti-inflammatory activity was expressed as percentage of NO production’s inhibition calculated with respect to control (untreated cells).

### 2.13. Anti-Candida Activity Testing

The antifungal activity against *Candida albicans* ATCC 10231 was evaluated by broth microdilution following the method reported by Andersen et al. [26]. *C. albicans* was grown aerobically in PDB_Glu_ at 37 °C. After 24 h, *Candida* suspension was diluted in PDB_Glu_ to reach a concentration of 4 × 10^5^ cells/mL (determined by counting in a Bürker chamber). The yeast suspension (50 μL) was inoculated in 96-well plates along with 50 μL of liposomal formulations (LP-Q, LP-GA and LP-Q+GA) or solutions of Q (in DMSO) and GA (in distilled water). Samples were diluted in a two-fold sequence to test concentrations ranging from 250 to 2 μg/mL. Plain-LP, DMSO and distilled water were used as negative, solvent, and growth control, respectively. Blank control, consisting only of growth medium, and sterility controls, containing formulations and sterile medium, were also included. Plates were incubated aerobically without shacking at 37 °C for 24 h. Afterwards, the growth inhibition was established through microscope observation (20×; Axiovert 40 Inverted Microscope, Carl Zeiss, Thornwood, NY, USA) and IC_50_ was defined as the minimal concentration of the sample that inhibits 50% or more of the visible growth, as compared to untreated control. Aliquots of the samples (20 μL) were spotted onto PDA_Glu_ and the minimal lethal dose (MLD) was defined as the concentration at which no growth was observed after 24 h of incubation at 37 °C.

All experiments were repeated in triplicate.

### 2.14. Statistical Analysis

All results were expressed as mean ± standard deviation (SD). For the comparison of two means, Student’s *t*-test was applied. One-way ANOVA followed by Bonferroni correction was used for multiple comparison. All statistical analyses were performed using GraphPad Prims version 8.0.0 for Windows (GraphPad Software, San Diego, CA, USA, www.graphpad.com) and differences were considered significant for *p* value < 0.05.

## 3. Results and Discussion

Vaginal drug administration for localized therapy is an interesting therapeutic choice considering the treatment of sexually transmitted diseases, fungal and bacterial infections, and cancer. The vaginal site is regarded as one of the most highly challenging sites for drug action and different approaches have been proposed as superior treatments in topical vaginal therapy. Conventional vaginal pharmaceutical forms are often associated with poor retention, low bioavailability, inability to modulate the release of drug and need for frequent administrations that reduce the patient compliance [6]. Smart nanocarrier-based drug delivery platforms offer possibilities to achieve sustained drug release, efficient cellular targeting, and even intrinsic antimicrobial properties [38,39].

Among nanocarriers, liposomes are particularly suitable for vaginal delivery because they are not interfering with vaginal microbiota and are able to protect active substances against external enzymatic degradation and rapid perturbations that can occur in the vaginal cavity [22]. Moreover, they are biodegradable, biocompatible, weakly immunogenic and non-irritating to vaginal mucosa. Their characteristics, such as composition, size and surface properties, will affect their fate in the vaginal site [23]. This study focused on the feasibility of simple phosphatidilcholine-based liposomes. Indeed, the entrapment of highly lipophilic molecules, such as Q, could be hampered by the presence of additional components in the lipidic bilayer. Considering that antifungal formulations need to act primarily on the vaginal epithelial surface, where *Candida* infection occurs, conventional liposomes could be a valid choice to obtain not-expensive and simple vesicles able to simultaneously accommodate two different active molecules.

### 3.1. Characterization of Liposomes

#### 3.1.1. Liposomal Size and Surface Properties

To optimize the loading of polyphenols, liposomal formulations varying in concentrations of Q and/or GA were prepared and characterized in terms of their size, PDI and zeta potential. Results are summarized in Table 1. Firstly, liposomes containing only Q were investigated.

Although extrusion is a widely used vesicle size reduction method [24,40], sonication was found more suitable in this work. When forced step-wise through polycarbonate membranes (0.8, 0.4 and 0.2 μm pore size filters, respectively) LP-Q precipitated, possibly because Q was expelled out of the lipid bilayer during the process, leading to unstable dispersions (mean size: 366.5 ± 9.2; PDI: 0.56 ± 0.01).

The starting concentrations of Q and/or GA are indicated in brackets. Formulations selected for further studies are presented in bold.

Considering the maximum Q load in vesicles, we observed that the size and PDI increased with the increasing starting concentration of Q (Table 1). In particular, when Q was added at the concentration of 2 mg/mL, liposomes were significantly bigger and the high PDI (>0.8) indicated the presence of precipitates. This was probably due to Q, being very poorly soluble in water, immediately precipitating when not incorporated in the lipid bilayers [41]. LP-Q prepared with the intermediate concentration (Q 1.5 mg/mL) were also excluded because of the stability issue; although equal in size to liposomes prepared with Q 1 mg/mL, they were not stable after just one week of storage at 4 °C (mean size: 504.6 ± 14.1; PDI: 0.564 ± 0.052).

On the contrary, liposomes formulated with GA in the aqueous phase at concentration of 2 mg/mL were stable over time, however when co-entrapped with Q 1 mg/mL, the resulting LP-Q+GA formulation precipitated.

For these reasons, the liposomal preparations LP-Q (Q 1 mg/mL), LP-GA (GA 1 mg/mL) and LP-Q+GA (Q 1 mg/mL; GA 1 mg/mL) were selected for further studies. They displayed an average diameter of around 200 nm that is estimated to be optimal for delivery to vaginal mucosa [31,42] and for targeting microorganisms able to grow biofilms [43], such as *Candida* spp. [44].

In particular, mean size of liposomes containing either only Q or GA did not differ significantly from plain-LP, while liposomes comprising both polyphenols (LP-Q+GA) were slightly bigger (*p* = 0.0012) as a consequence of the simultaneous incorporation of both polyphenols. Although sonication led to the formation of dispersions less homogeneous than those obtained by extrusion technique [40], results were reproducible and all PDI values were below 0.7 (0.348–0.438), considered acceptable for liposomal dispersions [45].

As previously observed [28,46], plain-LP exhibited a zeta potential close to neutral (Table 1) because phosphatidylcholine is a zwitter ionic lipid that can acquire slightly negative values in distilled water, as a result of the orientation of lipid headgroups and formation of a hydration layer around vesicles [47]. Liposomes incorporating Q and/or GA were more negative than plain-LP (*p* < 0.05), coherently with the fact that both Q and GA were negatively charged in water (−18.5 ± 0.6 mV and −14.3 ± 0.7 mV, respectively).

#### 3.1.2. Polyphenols Entrapment Efficiency

Despite the wide spectrum of beneficial properties exerted by Q [14], the low solubility of this molecule limits its wider therapeutic use. Moreover, chemical stability of Q is highly compromised in the presence of oxygen [48] and metal ions [49], as well as in alkaline conditions [50]. Among polyphenolic acids, GA exhibits several biological relevant activities [19,51]. However, its therapeutic potential is also hampered by low bioavailability [52] and susceptibility to environmental factors [53], such as the tendency to easily oxidize at pH above 7 [54] giving rise to metabolites, namely 4-O-methyl gallic acid and pyrogallol, that possess lower antioxidant activity compared to GA.

Due to their structure composed of phospholipid bilayers, liposomes can straightforwardly entrap both hydrophilic and lipophilic substances. In this regard, encapsulation in liposomal vesicles is expected to increase the physicochemical stability and local accumulation of active substances at the site of administration, and therefore the therapeutic efficiency of both polyphenols [55].

Co-encapsulation of active molecules in the same nanocarrier is particularly attractive because it allows the simultaneous delivery of molecules to their target, thus simplifying and improving the therapy. To date, Q was successfully co-encapsulated in liposomes with resveratrol [56] and epigallocatechin-3-gallate [57], whereas GA was co-loaded with resveratrol [58]. These formulations were mainly focused on the treatment of skin pathologies related to microbial infections or oxidative stress. To the best of our knowledge, no formulations comprising Q and GA have been proposed for localized vaginal treatment.

In Table 2 are reported the EE % of Q and GA formulated either as single polyphenol or in combination after 120 s of sonication (see Section 2.3).

Q was efficiently entrapped both in LP-Q and LP-Q+GA (EE % >85%), as a consequence of its lipophilic nature that favors its incorporation inside the lipid bilayer. This is in agreement with the reported high EE % for Q in liposomes, ranging from 67% to 89%, [56,59,60,61]. Recently, Riva et al. [41] also demonstrated that the employment of phytosomes can increase the solubility of this polyphenol, thus promoting the entrapment of Q into the vesicles.

The EE %, as well as size and PDI, of GA inside liposomes before and after different sonication times are reported in Table 3. The EE % of GA inside MLV was found to be 50.6 ± 0.6% and 47.5 ± 1.0% for LP-GA and LP-Q+GA, respectively. After sonication, the EE % decreased with increasing sonication time. Two minutes of sonication were required to obtain vesicles with the desired size; indeed, mean diameter of LP-GA was 600.1 ± 19.0 nm after 60 s of sonication and 314.8 ± 4.5 nm after 90 s, respectively, whereas LP-Q+GA had a size of 897.7 ± 5.4 nm after 60 s of sonication and 369.7 ± 4.7 nm after 90 s of sonication. Following 2 min of sonication the EE % was 25% for liposomes containing only GA and, interestingly, was higher for LP-Q+GA (30%, *p* = 0.0005) (Table 2). Our entrapment was higher than reported by Vitonyte et al. [58] for GA and resveratrol co-loaded liposomes. Considering its hydrophilic nature, GA was probably located inside aqueous core [57]. During ultrasound treatment, MLV undergo breakage and rearrangements that induce instability and formation of smaller vesicles. Our results suggest that the presence of Q in the surrounding lipid bilayer partially prevented leaks of GA from liposomes due to sonication process.

#### 3.1.3. Stability of Liposomal Preparations

**Figure 1:**
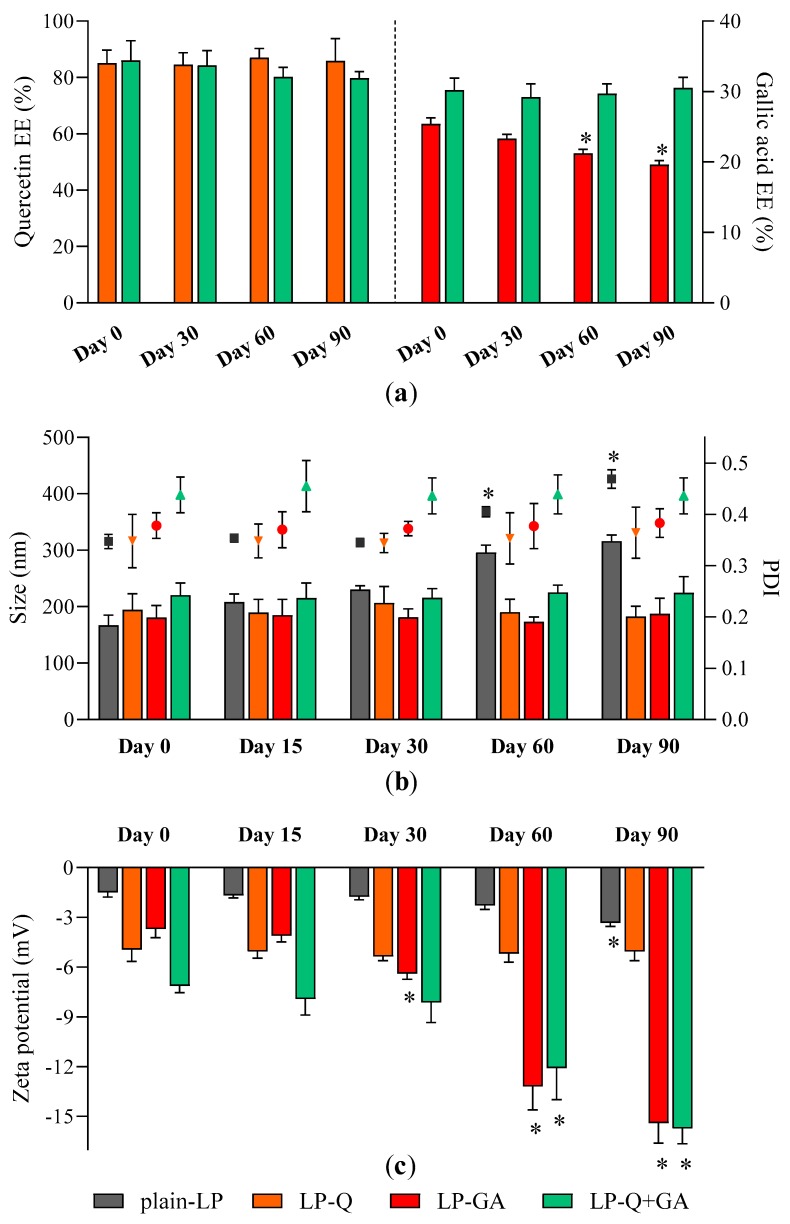
Stability of liposomes: changes in (**a**) Q and GA EE %; (**b**) liposomes size (bars, left Y axis) and PDI (symbols, right Y axis) and (**c**) zeta potential over a storage period of 90 days at 4 °C (mean ± SD, *n* = 4). The statistical significance was calculated with respect to day 0; * *p* < 0.05.

Storage stability of liposomal dispersions is an important aspect to predict the quality of formulations since the leakage of active molecules as well as aggregation should be avoided [62]. We evaluated stability by using entrapment efficiency (Figure 1a), particle size (Figure 1b) and zeta potential (Figure 1c) as parameters indicating changes from original liposomal characteristics.

No relevant changes in respect to size and PDI were detected for all formulations; moreover, polyphenol-containing liposomes appeared more stable than plain-LP (Figure 1b). As reported earlier by other authors [56,59,60], Q was effectively retained inside liposomes over the course of 3 months, with no significant loss and no signs of degradation. On the contrary, the EE % of GA formulated alone tended to slowly decrease after more than one month of storage indicating leakage from liposomes. After 90 days the EE % of LP-GA fell to 19.6 ± 0.6%, which corresponds to a decline of 22.8%, similarly to what was described by Manosroi et al. for GA encapsulated inside noisome formulations [55]. Notably, this phenomenon was not observed for liposomes comprising both Q and GA; those liposomes were able to prevent leakage of both polyphenols. Zeta potential of LP-GA and LP-Q+GA became more negative during storage while the surface charge of LP-Q was extremely stable. This evidence could be attributed to the tendency of GA to move and accumulate at the lipid bilayer–water interface. However, significant (*p* < 0.0001) decrease in zeta potential occurred after 30 days for LP-GA and after 60 days for LP-Q+GA.

#### 3.1.4. Release of Polyphenols from Liposomes

**Figure 2:**
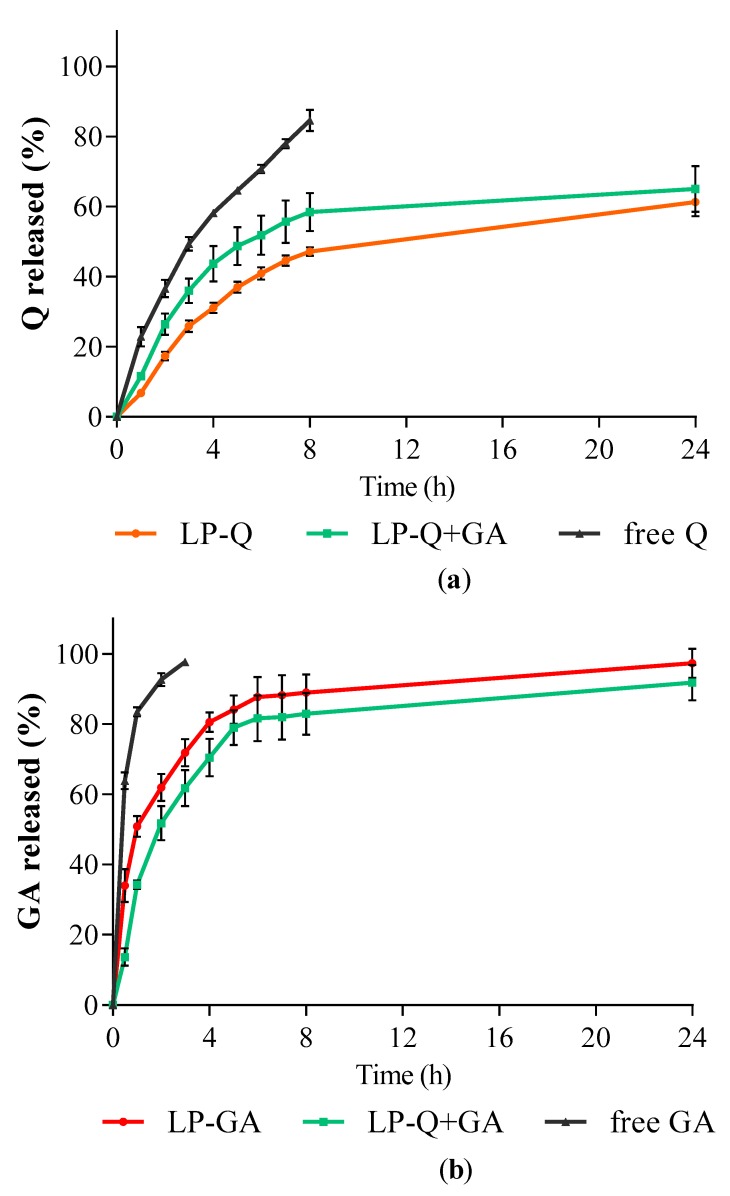
In vitro polyphenol release expressed as cumulative percentages of (**a**) Q and (**b**) GA released over time from liposomes containing only Q (LP-Q), liposomes containing only GA (LP-GA) and liposomes containing both polyphenols (LP-Q+GA) compared to free Q and free GA (mean ± SD, *n* = 3).

The in vitro release studies of polyphenols were performed by using Franz diffusion cells and phosphate buffer at pH 4.5 as receiving phase to mimic the acidity of a normal vaginal environment. The release behaviors of Q and GA from liposomes are depicted in Figure 2. Free Q and GA in solutions were used as controls for comparison. For all liposomal formulations, the cumulative release rates of polyphenols from liposomes were much slower compared to the respective controls (*p* < 0.001).

The release profiles of GA (Figure 2b) from both LP-GA and LP-Q+GA were biphasic, with an initial burst release in the first hour followed by a sustained release. Interestingly, significant differences in GA release rates were detected between LP-GA and LP-Q+GA at the earlier time points up to 4 h (*p* < 0.002). In particular, after 1 h, ~50% of GA was released from LP-GA and only ~34% from LP-Q+GA. After 8 h, both formulations released more than 80% of GA (*p* > 0.05) and release was complete within 24 h. The lipophilic nature of Q allowed a more evident sustained release of this polyphenol from liposomes (Figure 2a) [60]. About 47% of Q was released after 8 h from LP-Q. Notably, the co-presence of GA inside liposomes favored the release of Q (*p* < 0.03), and reached ~58% from LP-Q+GA after 8 h. Thus, the combination of two polyphenols not only extended the release of GA, guaranteeing a long-lasting activity at the site of administration, but also improved the release of Q required to elicit a stronger biological effect.

### 3.2. Biological Characterization

VVC is associated with increased levels of nitric oxide which leads to inflammatory response and consequent vulvar pain that can heavily impair the quality of life of many women worldwide [63]. Fidel et al. [5] also demonstrated that symptomatic, but not asymptomatic, women displayed high levels of polymorphonuclear leukocytes (PMN) responsible for release of pro-inflammatory mediators and free radicals in the vaginal lumen. The authors highlighted that recruitment of PMN was not only ineffective in protecting from fungus infections, but actually exacerbated VVC symptoms, including itching, burning and redness at the vulva and vaginal mucosa.

In this regard, polyphenols are attractive natural substances able to exert antioxidative and anti-inflammatory activities [10], minimizing the tissue damages and preventing the onset of chronic infections.

#### 3.2.1. Antioxidant Activity of Liposomal Polyphenols

**Figure 3:**
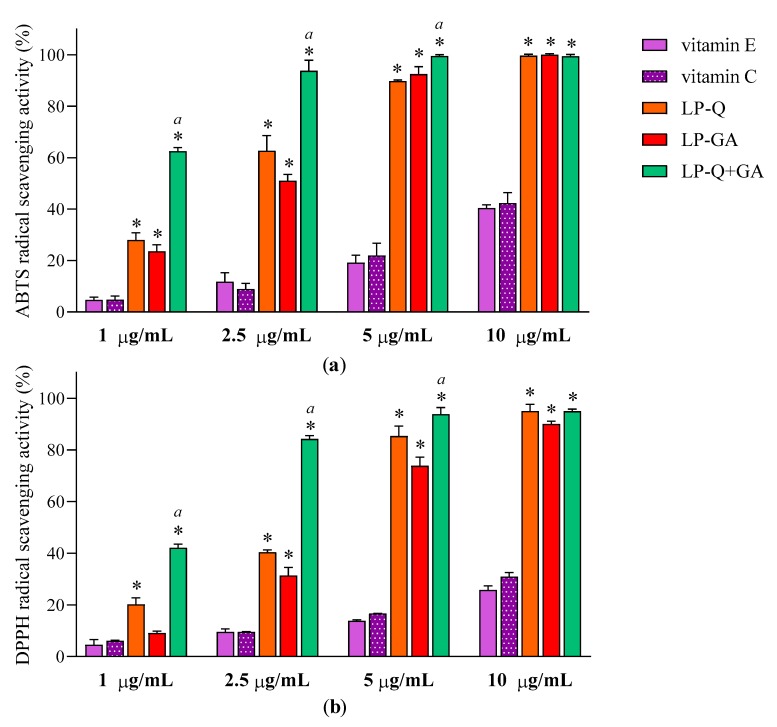
Antioxidant activities of LP-Q, LP-GA and LP-Q+GA, vitamin E and vitamin C expressed as (**a**) ABTS and (**b**) DPPH free radicals scavenging activity (mean ± SD, *n* = 3). The statistical significance with respect to vitamin E and vitamin C (used as comparison) was reported; * *p* < 0.0001. Statistical differences between liposomes holding both polyphenols (LP-Q+GA) and liposomes holding only one polyphenol (LP-Q/LP-GA) were also calculated; *a*: *p* < 0.001.

Antioxidant effect of polyphenols was evaluated after the complete dissolution of liposomal formulations in ethanol, by two colorimetric methods employed. Figure 3 reports the results at four different concentrations of Q and/or GA (1, 2.5, 5 and10 μg/mL) obtained for ABTS (Figure 3a) and DPPH assays (Figure 3b). With respect to well-known antioxidants (vitamin E and C), the two polyphenols exhibited significantly higher concentration-dependent scavenging activity against both free radicals (*p* < 0.0001), while plain-LP were ineffective, as expected. It is evident that Q and GA possessed strong antioxidant potentials; both were active also at very low concentrations (~11–31% at 1 μg/mL) and both ABTS**·**^+^ and DPPH radicals were almost completely inhibited at 10 μg/mL (~90–99%). This is in agreement with evidence reported in the literature for free Q [56] and free GA [64]. Considering that antioxidant activity plays a crucial role in the pharmacological effects of Q and GA, it is important to assure that their incorporation inside lipid nanocarriers and the entire preparative method did not compromise the capacity of the molecules to quickly and efficiently remove free radicals. It seems that the therapeutic activity of many polyphenols could be potentiated when co-administered with other polyphenols or antibiotics [65]. Recently, Q was reported to act in synergism with epigallocatechin-3-gallate [57] and with curcumin [65], enhancing the antioxidant and anti-inflammatory activities, respectively.

To investigate the effect of the combination of Q and GA, EC_50_ values were obtained from linear regression analysis (Table 4). The γ values, calculated according to the Equation (2) for ABTS and DPPH assays, were below 1, indicating that co-delivered polyphenols exerted an antioxidant activity in a synergistic way.

EC_50_ was defined as effective concentrations required for the 50% decrease of radicals in ABTS and DPPH assays (mean ± SD, *n =* 3).

#### 3.2.2. Anti-Inflammatory Activity of Free and Liposomal Polyphenols

It is well documented that both Q and GA are able to reduce inflammatory response in LPS-induced macrophages by acting at different levels of the inflammation cascade [19,66].

**Figure 4:**
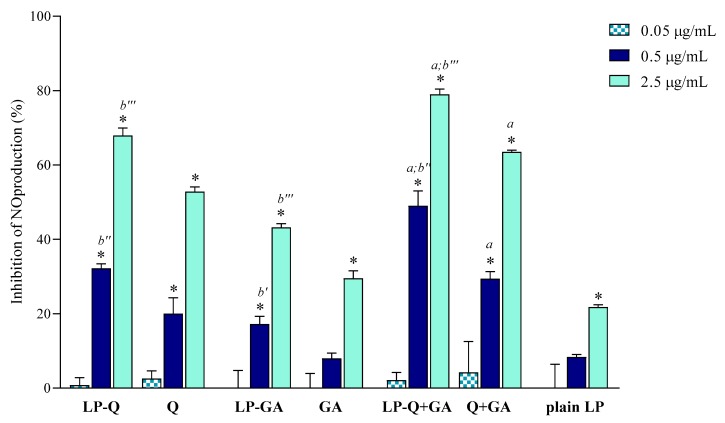
Inhibitory effect of liposomal and free Q and/or GA on nitric oxide (NO) production in lipopolysaccharide (LPS)-induced macrophages (mean ± SD, *n* = 3). The statistical significance was calculated with respect to control (untreated cells); * *p* < 0.0001. Statistical differences between coupled polyphenols (LP-Q+GA or Q+GA) and single polyphenol (LP-Q/LP-GA or Q/GA) were also investigated; *a*: *p* < 0.001. Statistical differences between liposomes and corresponding free polyphenols were reported as follows: *b′*: *p* < 0.01; *b″*: *p* < 0.001; *b‴*: *p* < 0.0001.

In the current work, we evaluated the inhibitory effects of liposomal polyphenols on the production of NO, a signaling molecule that plays a crucial role in the pathogenesis of inflammation and infections. LPS-induced macrophages were treated for 24 h with liposomal or free polyphenols at three different Q/GA concentrations (0.05, 0.5 and 2.5 μg/mL) (Figure 4). Plain-LP were also tested at the same SPC concentration as in liposomes containing polyphenols. No effect on NO production was observed at the lower concentration, but at 0.5 and 2.5 μg/mL polyphenol-containing liposomes displayed a strong concentration-dependent inhibition of NO production, that was significantly higher compared to plain-LP (*p* < 0.0001). A concentration-dependent behavior was also confirmed for free polyphenols. Notably, in all cases, the inhibitory effects on NO production were higher when Q and/or GA were incorporated inside liposomes (*p* < 0.009), in agreement with previous works on liposomal curcumin [37], resveratrol [46] and epicatechin [24] formulations. LP-Q seemed to be more potent with respect to LP-GA (inhibition of ~67% and ~43%, respectively, at 2.5 μg/mL) and the anti-inflammatory activity significantly increased (*p* < 0.0005) when the two molecules were delivered together (LP-Q+GA inhibited ~79% of NO production at 2.5 μg/mL), consistently with what observed for antioxidant activity.

Our results suggest that the choice of an appropriate delivery system improves the beneficial activity of polyphenols. Furthermore, combining Q and GA can be a promising strategy to take advantage of different mechanisms of action, thus heightening the overall anti-inflammatory effect and improving relief of pain associated with inflammation.

#### 3.2.3. Effect of Free and Liposomal Polyphenols on Cell Viability

**Figure 5:**
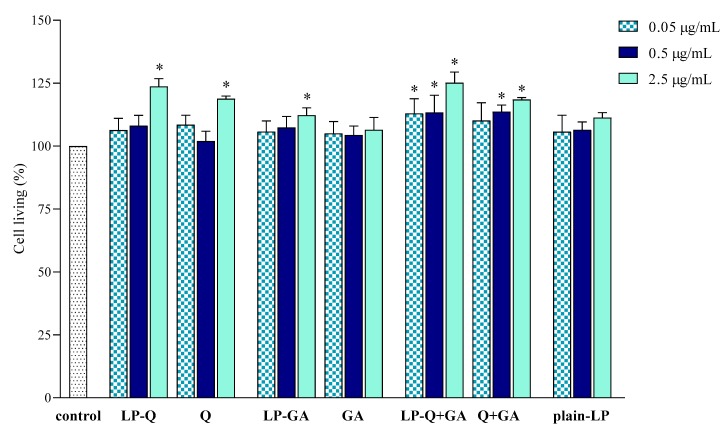
Effect of liposomal and free polyphenols on RAW 264.7 cell viability compared to viability of untreated cells (100%) (mean ± SD, *n* = 3). The statistical significance was calculated with respect to control; * *p* < 0.01.

The effect of liposomal formulations and free Q and/or GA on macrophage viability was also investigated and results are depicted in Figure 5. Cells were exposed to free and liposomal polyphenols for 24 h at the same concentrations tested for the anti-inflammatory activity. No cytotoxic effects were found.

LP-Q and LP-GA expressed weak mitogenic effects at the highest polyphenol concentration tested (2.5 μg/mL), that was also observed for LP-Q+GA at all concentrations.

These preliminary results indicated that both liposomal and free polyphenols were not toxic to living cells at the polyphenol concentration up to 2.5 μg/mL, corresponding to lipid concentration of 50 μg/mL. Moreover, the results suggest that the decreased NO production (Section 3.2.2) was actually due to the inhibition of inflammation and not to the inhibition of cell proliferation. The lack of cytotoxic action of Q on RAW 264.7 cell line and its ability to significantly inhibit NO production in LPS-induced macrophages was previously observed also by Mu et al. [67].

### 3.3. Antifungal Potential

Polyphenols not only possess useful biological properties, but also show few or no side effects and toxicity and thus may be promising natural molecules to challenge the problem of antifungal drug resistance. *Candida albicans* is the most frequent species isolated from women suffering from candidiasis and accounts for 80–90% of total vaginal fungal infections [1]. For this reason, the antifungal activity of polyphenol-containing liposomes and free polyphenols was evaluated against *C. albicans* ATCC 10231. Firstly, IC_50_ values were microscopically determined (Table 5) and, as expected, plain-LP made only from SPC had no effect [26]. Although Q is known to possess antibacterial activity [14], neither free Q nor LP-Q were active in inhibiting *Candida* growth.

On the contrary, the two liposomal formulations containing GA considerably hampered *Candida* growth in the concentration range between 31 and 63 μg/mL after 24 h of treatment (Figure S1 Supplementary Materials). Free GA displayed a similar behavior, indicating that liposomes effectively retained the antifungal activity of this phenolic acid. To better clarify the antifungal effect of liposomes, aliquots of samples exhibiting less than 50% growth were spotted onto agar plates and MLD determined after 24 h of incubation. Free GA, as well as liposomes comprising GA, showed fungicidal effects at concentrations higher than 125 μg/mL (Table 5), demonstrating that LP-GA and LP-Q+GA were efficient in counteracting *Candida* proliferation. Our findings were consistent with previous results obtained by Gehrke et al. [12] and Ozcelik et al. [13] on the GA activity against *C. albicans* ATCC 10231. The authors utilized either pure GA isolated from *Schinus lentiscifolius* extract or standard GA purchased from Sigma-Aldrich, respectively.

These promising findings could be further explored for localized treatment of infections and/or inflammation. By combining two or more polyphenols within a single nanocarrier, it would be possible to achieve efficient multitargeted treatment.

## 4. Conclusions

We have successfully developed innovative multi active-substance loaded liposomal formulations containing Q and GA, either as a single substance or in combination, for the treatment of vaginal diseases, in particular VVC. Liposomes containing both Q and GA had a desired size of about 200 nm and were able to encapsulate a high level of Q as well as favor the entrapment of GA. Moreover, they remained stable over time and provided a controlled release of both Q and GA.

LP-Q+GA displayed a strong antioxidant activity due to the synergism between the two entrapped polyphenols. Q and GA delivered together also exhibited the potent anti-inflammatory profile while remaining nontoxic.

Finally, LP-Q+GA efficiently inhibited the growth of *C. albicans*, the most frequent etiological agent of VVC. The effect could be attributed to the presence of GA and its fungicidal activity.

## Figures and Tables

**Table 1:** Liposomal characteristics: size, size distribution and zeta potential (mean ± SD, *n* = 4).

Type of Liposomes	Vesicle Size(nm)	PDI	Zeta Potential (mV)
plain-LP	166.9 ± 18.0	0.35 ± 0.14	−1.5 ± 0.2
LP-Q (Q 1 mg/mL)	194.4 ± 28.5	0.35 ± 0.05	−4.9 ± 0.7
LP-Q (Q 1.5 mg/mL)	194.6 ± 8.8	0.44 ± 0.01	−5.6 ± 0.2
LP-Q (Q 2 mg/mL)	594.3 ± 72.8	0.90 ± 0.01	−6.4 ± 0.3
LP-GA (GA 1 mg/mL)	180.7 ± 21.1	0.38 ± 0.03	−3.7 ± 0.5
LP-GA (GA 2 mg/mL)	289.4 ± 8.3	0.35 ± 0.08	−5.6 ± 0.2
LP-Q+GA (Q 1 mg/mL; GA 1 mg/mL)	220.4 ± 21.6	0.44 ± 0.04	−7.1 ± 0.4
LP-Q+GA (Q 1 mg/mL; GA 2 mg/mL)	366.5 ± 9.2	0.55 ± 0.02	−7.8 ± 0.7

**Table 2:** Entrapment efficiency of quercetin (Q EE %) and gallic acid (GA EE %) in the final formulations (liposomes sonicated for 120 s) (mean ± SD, *n* = 4).

Liposomes	Q EE %	GA EE %
LP-Q	85.1 ± 4.6	-
LP-GA	-	25.4 ± 0.9
LP-Q+GA	86.0 ± 7.0	30.2 ± 1.7

**Table 3:** Characteristics of liposomes containing GA before (multilamellar vesicles, MLV) and after different sonication times (60 s and 90 s): size, polydispersity index (PDI) and GA EE % (mean ± SD, *n* = 4).

Liposomes	Vesicle Size (nm)	PDI	GA EE %
LP-GA MLV	>1 μm	>0.9	50.6 ± 0.6
LP-GA sonicated-60 s	600.1 ± 19.0 nm	0.58 ± 0.09	36.8 ± 0.9
LP-GA sonicated-90 s	314.8 ± 4.5	0.44 ± 0.02	31.0 ± 0.7
LP-Q+GA MLV	>1 μm	>0.9	47.5 ± 1.0
LP-Q+GA sonicated-60 s	897.7 ± 5.4	0.64 ± 0.02	39.9 ± 1.5
LP-Q+GA sonicated-90 s	369.7 ± 4.7	0.56 ± 0.01	35.4 ± 0.7

**Table 4:** Antioxidant effect of polyphenol-containing liposomes.

Liposomes	ABTS Assay		DPPH Assay	
	EC_50_ (μg/mL)	γ	EC_50_ (μg/mL)	γ
LP-Q	1.61 ± 0.02	-	2.92 ± 0.17	-
LP-GA	2.49 ± 0.17	-	3.48 ± 0.03	-
LP-Q+GA	1.11 ± 0.06	0.79	1.54 ± 0.02	0.88

**Table 5:** Anti-*Candida* activity of polyphenols-containing liposomes reported as IC_50_ and minimal lethal dose (MLD) (mean ± SD, *n* = 3).

Liposomes	IC_50_ (μg/mL)	MLD (μg/mL)
LP-Q	no inhibition	
LP-GA	31–63	125
LP-Q+GA	31–63	125
plain-LP	no inhibition	
free Q	no inhibition	
free GA	31–63	125

## Supplementary Materials

The following are available online at https://www.mdpi.com/1999-4923/12/1/9/s1, Figure S1: Microscopically determination of anti-*Candida* activity.

## Abbreviations

ABTS	2,2-azino bis(3-ethylbenzothiazoline)-6-sulfonic acid diammonium salt;
DPPH	1,1-diphenyl-2-picrylhydrazyl;
GA	gallic acid;
LP	liposome;
LPS	lipopolysaccharide;
NO	nitric oxide;
PDI	polydispersity index;
Q	quercetin;
SPC	phosphatidylcholine;
VVC	vulvovaginal candidiasis
