# Supplementary material for: Utilizing Liposomal Quercetin and Gallic Acid in Localized Treatment of Vaginal Candida Infections
Source: Pharmaceutics. 2019 Dec 20;12(1):9. doi: 10.3390/pharmaceutics12010009 (PMC7023398; doi:10.3390/pharmaceutics12010009)
Supplement: Supplementary file 1 [file pharmaceutics-12-00009-s001.pdf]

pharmaceutics

Supplementary Materials: Utilizing Liposomal Quercetin and Gallic Acid in Localized Treatment of Vaginal *Candida* Infections

Barbara Giordani, Purusotam Basnet, Ekaterina Mishchenko, Barbara Luppi and Nataša Škalko-Basnet

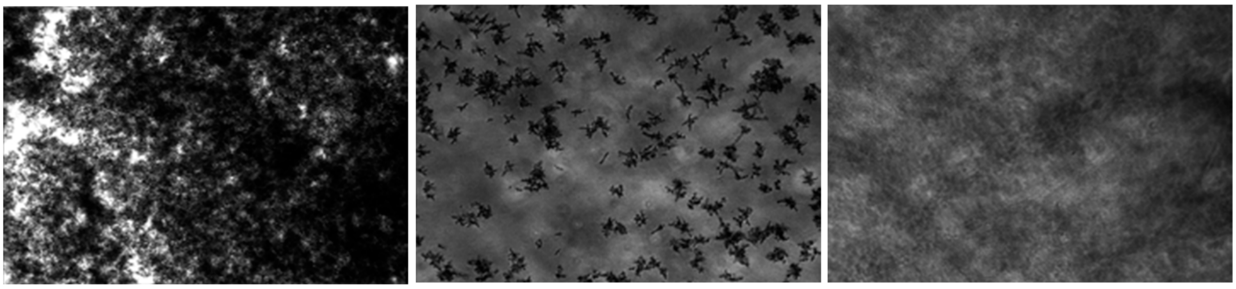

(**a**) (**b**) (**c**)

Figure S1. Microscopic determination of anti-Candida activity.Pictures of (**a**) Candida control (not treated cells); (**b**) Candida cells treated with GA 31 μg/mL and (**c**) Candidacell treated with GA 125 μg/mL were obtained using Axiovert 40 Inverted Microscope (20×).
